# LiverSCA 2.0: An Enhanced Comprehensive Cell Atlas for Human Hepatocellular Carcinoma and Intrahepatic Cholangiocarcinoma

**DOI:** 10.3390/cancers17050890

**Published:** 2025-03-05

**Authors:** Tina Suoangbaji, Renwen Long, Irene Oi-Lin Ng, Loey Lung-Yi Mak, Daniel Wai-Hung Ho

**Affiliations:** 1 State Key Laboratory of Liver Research, The University of Hong Kong, Hong Kong; u3009992@connect.hku.hk (T.S.); longrw@hku.hk (R.L.); iolng@hku.hk (I.O.-L.N.); 2 Department of Pathology, School of Clinical Medicine, The University of Hong Kong, Hong Kong; 3 Department of Medicine, School of Clinical Medicine, The University of Hong Kong, Hong Kong

**Keywords:** hepatocellular carcinoma, intrahepatic cholangiocarcinoma, metabolic dysfunction-associated steatotic liver disease/steatohepatitis

## Abstract

Background: Hepatocellular carcinoma (HCC) and intrahepatic cholangiocarcinoma (ICC) are two distinct types of primary liver cancer (PLC) characterized by considerable extents of cellular and molecular heterogeneities. We recently developed a web-based cell atlas called LiverSCA that possesses a user-friendly interface and comprehensive functionalities. It facilitates the exploration of gene expression patterns, cellular compositions, and intercellular communication within the microenvironments of liver and PLC tumors. Methods: To further enhance the documentation of data pinpointing different phenotypes/subtypes of liver and PLC, we extended the catalog of LiverSCA with additional datasets, e.g., ICC and metabolic dysfunction-associated steatotic liver disease/steatosis (MASLD/MASH). Results: The current enhanced version of the LiverSCA cell atlas encompasses six phenotypes (normal, HBV-HCC, HCV-HCC, non-viral HCC, ICC, and MASH), 63 patients, and over 248,000 cells. Furthermore, we have incorporated comparative visualization methods that allow users to simultaneously examine and compare gene expression levels between two different phenotypes. Conclusions: We are committed to the continuous development of LiverSCA and envision that it will serve as a valuable resource to support researchers in convenient investigations into the cellular and molecular landscapes of liver and PLC.

## Simple Summary

Primary liver cancer (PLC) is characterized by significant cellular and molecular heterogeneities. To aid research in this field, we developed LiverSCA, a web-based cell atlas with an intuitive interface that enables users to explore gene expression patterns, cellular compositions, and intercellular communication within liver and PLC tumor microenvironments. The catalog of the latest version of LiverSCA was expanded by including additional datasets, such as intrahepatic cholangiocarcinoma (ICC) and the livers of metabolic dysfunction-associated steatotic liver disease (MASLD/MASH). It now features six phenotypes (normal, HBV-HCC, HCV-HCC, non-viral HCC, ICC, and MASH liver), encompassing data from 63 patients and >248,000 cells. New comparative visualization methods allow users to examine gene expression levels between different phenotypes, making LiverSCA a vital resource for researchers investigating the cellular and molecular landscapes of PLC.

## 1. Introduction

Primary liver cancer (PLC) largely includes hepatocellular carcinoma (HCC) and intrahepatic cholangiocarcinoma (ICC), with HCC being the most prevalent version, accounting for more than 90% of all PLC cases [1]. The increase in incidence and mortality rate of HCC is primarily attributed to the rising rates of chronic liver diseases, particularly those caused by hepatitis B viruses (HBV) and hepatitis C viruses (HCV), as well as alcohol-related liver disease and metabolic dysfunction fatty liver disease (MAFLD) [2]. Furthermore, the progression of HCC is a complex multistep process, including chronic inflammation, fibrosis, and cirrhosis, eventually leading to malignancy. Several etiological factors of HCC have been identified, and specific molecular and immune classes have been identified for cancer development [3,4]. Similarly, ICC mostly develops in a background of chronic inflammation, which causes cholestasis and subsequent cholangiocyte injury [5], and is a subtype of cholangiocarcinoma (CCA), which encompasses a diverse group of malignancies affecting the biliary tree [6]. Based on the anatomical site of origin, CCA can be classified into two additional subtypes: perihilar (pCCA) and distal (dCCA) CCA [7]. The anatomical distinction between pCCA and dCCA lies in the insertion of the cystic duct, allowing both to be collectively referred to as ’extrahepatic’ (eCCA) [8]. HBV-HCC, HCV-HCC, and ICC exhibit significant cellular and molecular heterogeneities, reflecting their distinct etiologies, pathogenesis, and tumor microenvironment (TME). HBV-HCC arises from hepatocytes and is primarily driven by chronic viral hepatitis, leading to inflammation and cirrhosis. HBV infection drives HCC through direct and indirect mechanisms [9]. Directly, HBV integrates into or mutates host DNA, causing genomic instability, epigenetic changes, and abnormal expression of oncogenes and tumor suppressor genes. It also activates cancer-related pathways and alters cell metabolism, promoting malignant transformation. Indirectly, HBV-induced chronic inflammation and immune cell interactions create a pro-tumor microenvironment. The virus evades immune surveillance, allowing disease progression from inflammation to tumor formation. These combined effects enable HBV to disrupt normal liver function and drive HCC. In contrast, HCV-HCC also originates from hepatocytes but lacks viral DNA integration. Instead, HCV-induced chronic inflammation, oxidative stress, and liver fibrosis create a pro-tumorigenic environment [10]. HCV proteins exacerbate these processes, leading to mutations in genes like TP53 and CTNNB1, which are commonly altered in HCC. Unlike HBV-HCC and HCV-HCC, ICC originates from cholangiocytes, the epithelial cells lining the bile ducts. ICC is characterized by distinct genetic alterations, including mutations in KRAS, IDH1/2, and FGFR2 fusions, as well as epigenetic changes and dysregulated bile acid metabolism [6]. These molecular differences underscore the unique pathogenesis of ICC compared to HCC. Currently, the poor outcomes associated with HCC and ICC have been dramatically changing due to the advent of effective systemic therapies, including immune-checkpoint inhibitors (ICIs), tyrosine kinase inhibitors (TKIs), and their combined usage [3,11]. In recent years, due to advances in high throughput next-generation sequencing technologies, particularly in single-cell transcriptomics, the concept of a web-based cell atlas has acquired development momentum, which serves as a digital resource that catalogs and characterizes various cell types from different organisms and tissues and helps wet-lab researchers to conveniently access cellular and molecular data without bioinformatic skills. Nevertheless, there are not many publicly available cell atlases regarding liver tissue, and that of PLC tissue is particularly pauce [12,13]. More importantly, regarding existing versions, most of them merely provide limited and elementary functions, such as gene expression visualization of a single gene or functional enrichment. To this end, we recently developed a cell atlas called LiverSCA [14], aiming to facilitate the comprehensive data exploration of the intricate cellular and molecular landscapes of the liver and HCC tumor microenvironment. In our cell atlas, we included single-cell RNA sequencing (scRNA-seq) data for 40 tumor tissue samples collected from 35 HCC patients with distinctive etiological risk factors (HBV, HCV, and non-viral) and five healthy liver tissue samples. Using data from more than 147,000 cells after quality control filtering included in LiverSCA, it annotated and provided gene expression profiles of normal and malignant hepatocytes, liver sinusoid endothelial cells (LSECs), fibroblasts, and immune cells at single-cell resolution. For the functional modules in our cell atlas, we offered seven major functionalities, including differently expressed gene detection, functional enrichment, cellular communication, and pseudo-time trajectory inference, as well as a variety of visualization methods that allow gene expression examination regarding a single gene or a set of genes. In comparison with other existing tools in liver cancer, LiverSCA has unique and more comprehensive functionalities that allow the stratification and comparison of data regarding etiological risk factors that distinguish itself from the others. We have committed to the continuous development of LiverSCA and envisioned the increasingly important focus on ICC and MASLD/MASH. In this updated version, we incorporated a major update on LiverSCA by adding new data on ICC and MASLD/MASH. Currently, it consists of data regarding the phenotypes of PLC (HBV-HCC, HCV-HCC, non-viral HCC, and ICC) and liver (normal and MASLD/MASH). Moreover, LiverSCA also provides new functionalities, facilitating the comparison between phenotypes.

## 2. Materials and Methods

### 2.1. Sample and Data Processing

LiverSCA v2 was built on the basis of v1 (existing samples: 40 cases and total cell count of 147,142 cells) [15,16,17,18,19]. In this update, additional scRNA-seq data were collected from eight human ICC samples of two reported studies. In particular, the data of five ICC patient samples were sourced from Zhang et al. [20], comprising four treatment-naïve samples and one recurrent sample. Data from three additional ICC samples were acquired from Ma et al. [21]. Given the lack of accessible MASH-related human HCC samples, we could only procure two MASH-related non-cancerous samples, which had reached either MASH or fibrosis stages. MASH-stage liver samples were collected from patients undergoing bariatric surgery [22], whereas fibrosis-stage liver samples were obtained from patients undergoing orthotopic liver transplantation [23]. In the previous version of LiverSCA, we only provided data on three etiologies of HCC: HBV, HCV, and non-viral, along with healthy liver samples (Supplementary Table S2). Non-viral HCC refers to the cases that occur in the absence of evidence for viral infections, such as HBV or HCV, and without documented association with other well-defined risk factors (we cannot rule out the possibility of different non-viral etiologies as most studies only simply reported viral infection status). Taken together, we integrated different data into our cell atlas to represent a comprehensive collection of phenotypes and risk factors throughout the course of PLC.

### 2.2. Quality Control of Data

To ensure the integrity and reliability of the analysis, we implemented strict quality control measures for the dataset. Specifically, we excluded cells that exhibited a low library size, defined as those with fewer than 800 unique molecular identifiers (UMIs). Additionally, we removed cells with a high percentage of mitochondrial genome representation, specifically those exceeding 10%. This step is crucial, as a high mitochondrial content can indicate compromised cell condition, e.g., damaged or dying cells. Furthermore, we excluded genes with low detection levels of UMI count (less than 200). By applying these criteria, we aimed to enhance the quality of the data and ensure that subsequent analyses would yield more accurate and meaningful results.

### 2.3. Batch Effect Correction

We utilized the Harmony algorithm [24], a state-of-the-art tool specifically designed for integrating multiple datasets while preserving biological variability to address and remove undesirable technical artifacts arising from data batches or sources. The Harmony algorithm operates by aligning datasets in a low-dimensional space, ensuring that batch effects are minimized without compromising the underlying biological signals. The Harmony algorithm was implemented through the RunHarmony function from the Harmony R package, which seamlessly integrates with single-cell RNA sequencing analysis workflows. To assess the effectiveness of batch effect correction, we compared cell clustering patterns before and after applying the Harmony algorithm, observing significant improvements in the alignment of cells across batches. Following batch correction, we conducted further analyses on the harmonized dataset, including differential expression analysis to identify genes with significant changes, cell type identification to classify distinct populations, and cellular crosstalk analysis to explore interactions between cell types. After successfully removing technical variations among cohorts, the datasets were consolidated into respective Seurat objects, a widely used framework for single-cell data analysis, and organized according to their phenotypic characteristics. This approach ensured robust and reproducible downstream analyses, enabling us to draw meaningful biological insights from integrated datasets while mitigating the confounding effects of batch-related noise.

### 2.4. Website Building

The LiverSCA is based on a modular architecture with independent components. The backend is written in Python3 and is mainly responsible for manipulating the data and processing the user’s requests. The user-friendly interactive frontend is generated by a lightweight Javascript library and in-house scripts. A MySQL database engine is used to store meta information and parameters. This design makes LiverSCA scalable and enables efficient resource management.

## 3. Results

### 3.1. Overview of New Data

**Figure 1:**
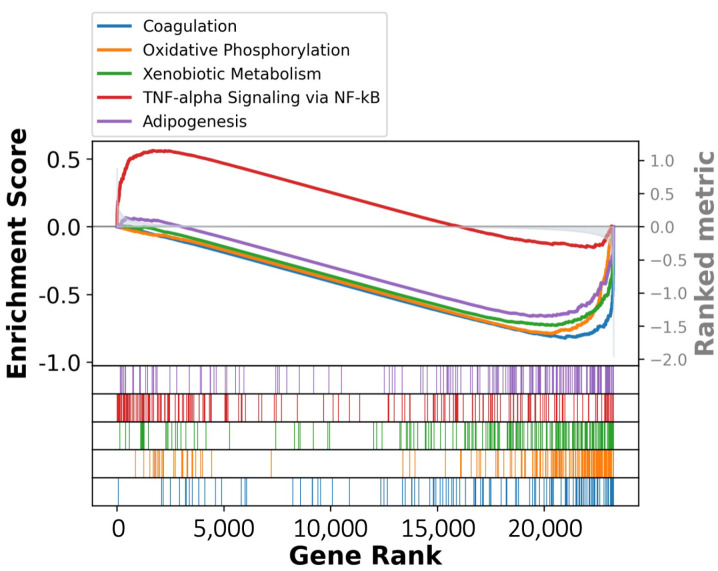
Top five MSigDB hallmark signatures identified through GSEA in the T cells of MASH compared to normal liver.

To incorporate a broader range of etiological risk factors for PLC into our cell atlas, in this update, we incorporated new scRNA-seq data corresponding to two additional phenotypes (ICC and MASH) (Supplementary Table S1). The dataset of ICC, which was derived from eight samples, comprises over 40,000 cells. It has not undergone cell sorting in sample preparation, and it contains both parenchymal (cholangiocyte) and non-parenchymal (stromal and immune) cells (Table 1). On the other hand, the MASH dataset was subjected to cell sorting for immune cell enrichment, particularly T cells. Hence, due to this immune cell selection process in constructing the MASH dataset, we recommend only using it to perform differential gene expression and functional enrichment analyses instead of cellular composition comparison. Regarding the T cells, those identified in the MASH samples were enriched in the TNF-alpha signaling pathway when compared to those in the normal samples, indicating an inflammation-related immune response due to the MASH disease background (Figure 1). In summary, the current enhanced version of the LiverSCA cell atlas encompasses six phenotypes (normal, HBV-HCC, HCV-HCC, non-viral HCC, ICC, and MASH), 63 patients, and over 248,000 cells (Table 1 and Supplementary Table S2).

### 3.2. Comparison of Four Different PLC Phenotypes

**Figure 2:**
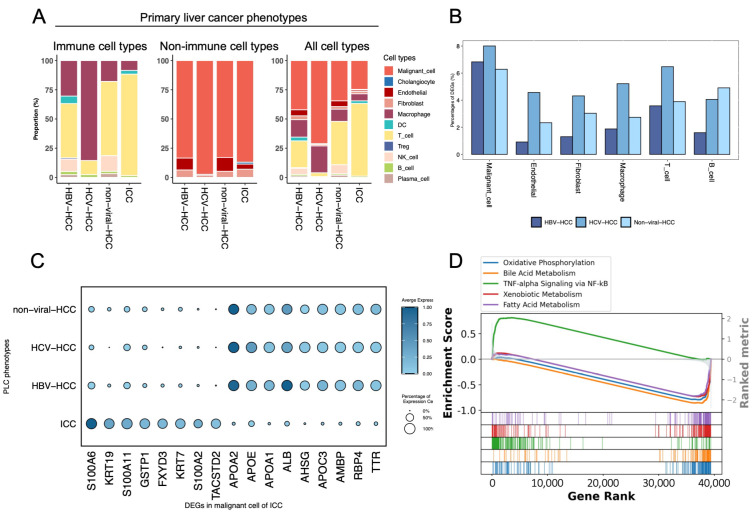
Summary of PLC datasets in LiverSCA. (**A**). Comparison of cell type composition among various phenotypes of primary liver cancer (PLC), categorized by immune cell types, non-immune cell types, and the total cell population. The x-axis represents the four phenotypes of PLC: HBV, HCV, and non-viral HCC and ICC; the y-axis indicates the percentage of each cell type within these phenotypes. (**B**). Proportion of significant dysregulated genes in each cell type of ICC compared to three types of HCC, respectively. (**C**). The dot plot displays the expression levels of several DEGs in malignant cells of ICC compared to the three HCC phenotypes. The y-axis represents the malignant cells across the four PLC phenotypes. (**D**). Top five MSigDB hallmark signatures identified through GSEA in malignant cells of ICC compared to HCC.

In this update, LiverSCA contains PLC data for multiple subtypes (HBV, HCV, non-viral, and ICC), enabling us to explore the cellular and molecular variations among them. Each of the four PLC phenotypes exhibited varying proportions of tumor-infiltrating immune cells in the tumor microenvironment (TME). In comparison to the other three hepatic cancer conditions, the TME of HCV demonstrated a higher existence of tumor-associated macrophages (TAMs), whereas ICC and the other two types of HCC exhibited a greater proportion of T lymphocytes (Figure 2A). Echoing our observations, Song et al. [25] found a higher enrichment of TAMs in the HCV-associated TME compared to that of HBV, suggesting that HCV-associated HCC patients may exhibit less susceptibility to immunotherapies due to the immunosuppressive effects of TAMs potentially limiting T cell immunity and, hence, the effectiveness of therapies. Upon examining the proportions of differentially expressed genes in the three subtypes of HCC (HBV, HCV, and non-viral), with ICC as reference, we consistently identified that the most contrasting cell type between the two predominant forms of PLC (HCC vs ICC) was malignant cells, indicating the significant extent of intrinsic differences between them (Figure 2B). Consequently, we analyzed the dysregulated genes in the malignant cells across the four PLC phenotypes and discovered that several S100 family genes (S100A6, S100A11, and S100A2) were significantly upregulated in the malignant cells of ICC, whereas they were not in the other subtypes of HCC. In contrast, apolipoprotein genes, including APOA, APOC, and APOE, exhibited higher expression levels in the malignant cells of HCC (Figure 2C). Gene set enrichment analysis (GSEA) revealed that when compared to those in HCC, the malignant cells in ICC were significantly enriched in the TNF-alpha signaling pathway (Figure 2D).

### 3.3. Comparative Visualization Approaches

**Figure 3:**
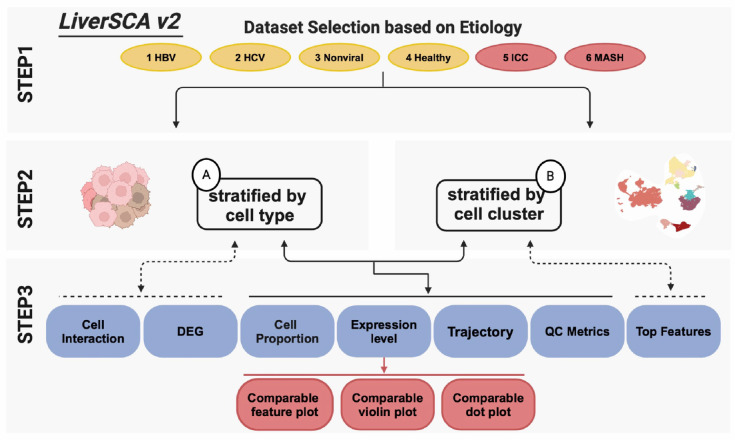
Overview of updated LiverSCA cell atlas. STEP1 displays six phenotypes; yellow represents four phenotypes in the previous version, and red represents updated phenotypes in the current version. The dashed lines indicate the only available options for the corresponding stratification method in Step 2. The red frames in STEP3 represent the updated sections for comparison visualization.

**Figure 4:**
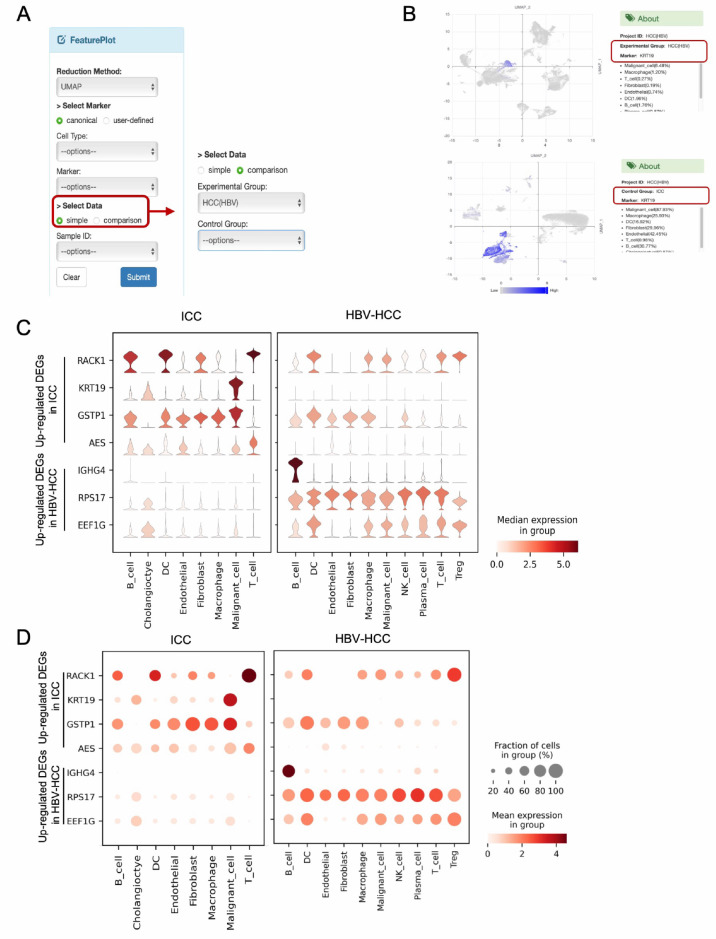
Illustrations of the updated comparative visualization methods. (**A**). Screenshot of the option to perform comparative visualizations in the FeaturePlot function model. The default option (**left**), and change to comparative visualizations (**right**). (**B**). Example of using the comparative FeaturePlot to check the expression of KRT19 in HBV-HCC (**upper**) and ICC (**lower**). (**C**,**D**). Comparative violin plots and dot plots illustrating the expression profiles of selected genes in two selected phenotypes, simultaneously.

The previous version of LiverSCA lacked a visualization method that enables the simultaneous examination of gene expression levels between two distinct phenotypes, e.g., HBV-HCC vs. HCV-HCC. To address this limitation, the current version allows three comparative visualization approaches: feature plots, violin plots, and dot plots (Figure 3). In the FeaturePlot module, users can select a single marker based on a canonical approach that displays a set of genes used to define the corresponding cell type or according to a user-defined criterion. Following marker selection, users can utilize the comparison option within the SelectData function, which displays the experimental groups corresponding to the initial phenotype selections, as well as a control group, allowing users to choose any group of interest (Figure 4A). Ultimately, the expression of the selected marker can be illustrated in two separate feature plots (Figure 4B). In the VlnPlot module, a similar method to that in the FeaturePlot is employed for marker selection; however, instead of a single marker, this module permits the selection of multiple markers simultaneously. The visualization results are presented as two separate panels of violin plots and dot plots based on the selected phenotypes. For example, comparative violin plots and dot plots can be used to illustrate the expression of dysregulated genes between ICC and HBV-HCC (Figure 4C,D).

## 4. Discussion

The molecular and clinical characteristics of HCC and ICC are distinct; however, they share overlapping risk factors and common pathways in oncogenesis [26]. Chronic inflammation is a hallmark of PLC phenotypes, determined by persistent viral infection in HBV-HCC and HCV-HCC and by biliary injury in ICC [27]. Chronic inflammation drives liver carcinogenesis by creating a cycle of cell death and regeneration. This process generates signals that promote cell survival and proliferation, leading to the formation of regenerative nodules, dysplasia, and ultimately cancer [28]. In this context, the phenotype of PLC may be shaped by the interplay between oncogenic drivers and the immune microenvironment, highlighting the critical role of immune-tumor interactions in PLC development. Epigenetic modifications, including DNA methylation and histone acetylation, also play a significant role in the development and progression of these cancers [29]. Furthermore, the TME in PLC phenotypes is characterized by immune cell infiltration, stromal remodeling, and hypoxia, which collectively influence tumor behavior and response to therapy. However, the composition and functional role of immune cells within the TME varies significantly among these phenotypes, contributing to their distinct clinical behaviors and therapeutic responses.

The findings presented by LiverSCA provide valuable insights into the cellular and molecular heterogeneities among different phenotypes of PLC, including HBV-HCC, HCV-HCC, non-viral HCC, and ICC. By leveraging the updated version of LiverSCA, researchers have been able to explore the TME and gene expression profiles across these phenotypes, revealing distinct immune cell compositions and molecular signatures. One of the key observations is the differential immune cell infiltration patterns among the PLC phenotypes; for instance, the TME of HCV-HCC exhibited a higher proportion of TAMs compared to other phenotypes. This finding aligns with previous studies, such as those by Song et al. [25], which have highlighted the immunosuppressive role of TAMs in HCV-HCC. In the specific immune microenvironment of hepatic neoplasms, TAMs exhibit a variety of functions, including the regulation of signal-transduction mechanisms, the change in self-metabolism patterns in response to the context of the internal environment, and the spectrum of secreted factors [30]. In contrast, ICC and other HCC phenotypes (HBV and non-viral) showed a greater proportion of T lymphocytes, suggesting a potentially more immunogenic TME that might be more responsive to immunotherapeutic interventions. These findings underscore the importance of considering the immune context of the TME when designing personalized treatment strategies for PLC patients. At the molecular level, LiverSCA has revealed significant differences in gene expression profiles between HCC and ICC, particularly in malignant cells. For example, ICC malignant cells exhibited upregulation of S100 family genes, such as S100A6, S100A11, and S100A2. These genes are involved in various cellular processes, including inflammation, proliferation, and metastasis, and their overexpression in ICC may contribute to its aggressive clinical phenotype. Zhang et al. reported that S100A11 is an independent prognostic factor of ICC and depends on the activation of the P38/MAPK signaling pathway to promote ICC cell proliferation [31]. Conversely, HCC malignant cells show higher expression of apolipoprotein genes, such as APOA, APOC, and APOE, suggesting distinct metabolic reprogramming in HCC compared to ICC. These findings highlight the importance of phenotype-specific molecular characterization in understanding the underlying biology of PLC and identifying potential therapeutic targets.

The updated version of LiverSCA significantly enhanced the comprehensiveness and functionality of the cell atlas by introducing a new analysis of gene expression using multiple comparative visualization plots and including a more comprehensive collection of datasets that pinpoint the different subtypes or etiological risk factors of PLC. By using LiverSCA, the ability to visualize single-cell data in a user-friendly manner can facilitate collaboration between computational and wet-lab researchers, fostering a more comprehensive understanding of PLC. Moreover, the observation that TMEs differ significantly across PLC subtypes/phenotypes, with variable immune cell infiltration patterns, likely advocates the need for more specialized therapeutic approaches pinpointing the intrinsic properties of PLC.

Regarding the future development of LiverSCA, we will continue to strive for enhancement by adding more etiologically-specific PLC datasets. It is envisioned that LiverSCA can serve as a vital resource for researchers aiming to elucidate the complexity and heterogeneity of PLC. We hope future updates of LiverSCA can include an even larger collection of liver- and PLC-related phenotypes.

Due to the limited availability of human HCC samples associated with MASH, our LiverSCA cell atlas was restricted to two non-malignant samples that had progressed to either the MASH or fibrotic stage. Although the current patient cohort is not large, it encompasses a variety of common etiologies of PLC, which may provide valuable insights despite the limited sample size.

## 5. Conclusions

In summary, we believe LiverSCA is currently one of the most comprehensive and well-maintained liver- and PLC-related cell atlases. Our future endeavors will continue to work towards developments that pinpoint the addition of new sample cohorts, enhance the cell count, and incorporate more comprehensive data analysis and visualization functionalities. Moreover, our exploration of the latest LiverSCA version signified the importance of distinctive immune cell composition among different subtypes of PLC, particularly the enrichment of TAMs in HCV-HCC and the differential expression of key genes, such as the S100 family and apolipoproteins; this may also contribute to the varying clinical behaviors and therapeutic responses observed in PLC.

## Figures and Tables

**Table 1:** Summary of cell counts in each cell type of all samples in LiverSCA.

Etiology	Sample ID	No. Patients	Tumor Cell	Hepato-Cyte	Cholan-Giocyte	LSEC	Fibro-Blast	Macro-Phage	Dendritic Cell	T Cell	NK Cell	B Cell	Plasma Cell	Total Cell Count
ICC	ICC_N1	5	9464	0	184	354	393	1476	260	651	0	172	0	12,954
	ICC_N2	3	934	0	0	169	448	1085	674	25,505	0	283	0	29,098
MASH	MASH_N1	10	0	13	0	35	0	3799	84	27,935	17,771	2526	192	52,355
	MASH_N2	5	0	138	0	257	35	1336	28	3503	961	505	130	6893
Healthy	healthy_N1	5	0	3732	0	817	40	1191	0	1185	843	127	503	8438
HBV	HBV_N1	1	2598	0	0	853	437	845	58	97	3	16	2	4909
	HBV_N2	5	4818	0	0	1149	883	3300	466	3851	1174	381	364	16,386
	HBV_N3	9	1135	0	0	2119	1257	431	196	2144	908	65	191	8446
	HBV_N4	9	25,578	0	0	42	10	7607	1826	12,952	2180	618	361	51,174
HCV	HCV_N1	2	5249	0	0	44	95	1703	0	240	0	47	0	7378
Non_viral	non_viral_N1	1	11,165	0	0	743	475	510	0	71	0	1	8	12,973
	non_viral_N2	3	2518	0	0	584	493	2315	0	1441	199	51	119	7720
	non_viral_N3	5	3758	0	0	1173	119	2259	0	17,315	3744	502	838	29,718

## Data Availability

LiverSCA is freely accessible using the following link: https://patholiver.hku.hk/liverp/, accessed on 2 March 2025.

## Supplementary Materials

The following supporting information can be downloaded at: https://www.mdpi.com/article/10.3390/cancers17050890/s1. Table S1. Summary of cell counts in each cell type of updated samples in LiverSCA. Table S2. Clinical and pathological characteristics of the samples in LiverSCA.

## Abbreviations

The following abbreviations are used in this manuscript:

PLC	Primary liver cancer
HCC	Hepatocellular carcinoma
ICC	Intrahepatic cholangiocarcinoma
MASLD	Metabolic dysfunction fatty liver disease
HBV	Hepatitis B viruses
HCV	Hepatitis C viruses
ICIs	Immune-checkpoint inhibitors
TKIs	Tyrosine kinase inhibitors
ScRNA-seq	Single-cell RNA sequencing
LSECs	Liver sinusoid endothelial cells
TME	Tumor microenvironment
TAMs	Tumor-associated macrophages
GSEA	Gene set enrichment analysis
