# Supplementary material for: LiverSCA 2.0: An Enhanced Comprehensive Cell Atlas for Human Hepatocellular Carcinoma and Intrahepatic Cholangiocarcinoma
Source: Cancers (Basel). 2025 Mar 5;17(5):890. doi: 10.3390/cancers17050890 (PMC11898674; doi:10.3390/cancers17050890)
Supplement: Supplementary file 1 [file cancers-17-00890-s001.zip › cancers-3453783-supplementary.pdf]

Table S1. Summary of cell counts in each cell type of updated samples in LiverSCA.

**Sample ID**	**Etiology**	**Malignant cell**	**Hepatocyte**	**Cholangiocyte**	**Endothelial**	**Fibroblast**	**Macrophage**	**Dendritic cell**	**T cell**	**NK cell**	**B cell**	**Plasma cell**	**Total cell count**
ICC_N1	ICC	9464	0	184	354	393	1476	260	651	0	172	0	12954
ICC_N2		934	0	0	169	448	1085	674	25505	0	283	0	29098
MASH_N1	MASH	0	13	0	35	0	3799	84	27935	17771	2526	192	52355
MASH_N2		0	138	0	257	35	1336	28	3503	961	505	130	6893

Table S2. Clinical and pathological characteristics of the samples in LiverSCA.

**Phenotype**	**Sample ID**	**Accession number**	**Gender**	**Age**	**Clinical Info***	**Sample Info**	**Cell Count**
HBV-HCC	HBV_N1	GSE112271	Female	66	Moderately[1]	Sequenced four tumor regions	4909
	HBV_N2	GSE149614	Male	66	T1N0M0[2]	Each patient collected one primary tumor sample	16386
			Male	65	T1N0M0[2]		
			Female	64	T3N0M0[2]		
			Male	48	T4N0M0[2]		
			Male	53	T4N0M1[2]		
	HBV_N3	GSE156625	Male	59	NA	Tumor tissue from multiple sectors	8446
			Female	71			
			Male	75			
			Male	68			
			Male	65			
			Male	77			
			Male	77			
			Male	55			
			Male	56			
	HBV_N4	PRJNA932937	Male	74	III[3]	Each patient collected one primary tumor sample	51174
			Male	45	III-IV[3]		
			Male	77	II-III[3]		
			Male	54	III-IV[3]		
			Male	61	III-IV[3]		
			Female	73	III-IV[3]		
			Male	55	III[3]		
			Male	61	III-IV[3]		
			Male	65	III[3]		
HCV-HCC	HCV_N1	GSE149614	Male	66	T1N0M0[2]	Each patient collected one primary tumor sample	7378
				60	T2N0M0[2]		

Non-viral-HCC	non_viral_N1	GSE112271	Male	67	Moderately[1]	Sequenced three tumor regions	12973
	non_viral_N2	GSE149614	Male	65	T3N0M0[2]	Each patient collected one primary tumor sample	7720
			Male	48	T4N0M0[2]		
			Male	64	T4N0M0[2]		
	non_viral_N3	GSE156625	Male	74	NA	Tumor tissue from multiple sectors	29718
			Female	72			
			Female	48			
			Male	56			
			Male	76			
ICC	ICC_N1	GSE138709	Female	NA	Poorly[1]	Treatment-naïve ICC samples	12954
			Female		Moderately[1]		
			Male		Poorly[1]		
			Male		Moderately[1]		
			Male		Poorly[1]		
	ICC_N2	GSE189903	NA	NA	NA	Tumor tissue from multiple sectors	29098

NA: Not available

*clinical info contains several systems to define HCC and ICC stages: 1: tumor grade; 2: TNM staging; 3: Edmondson grading
